# Halotropism requires phospholipase Dζ1‐mediated modulation of cellular polarity of auxin transport carriers

**DOI:** 10.1111/pce.13646

**Published:** 2019-10-02

**Authors:** Ruud A. Korver, Thea van den Berg, A. Jessica Meyer, Carlos S. Galvan‐Ampudia, Kirsten H.W.J. ten Tusscher, Christa Testerink

**Affiliations:** ^1^ Plant Physiology and Cell Biology, Swammerdam Institute for Life Sciences University of Amsterdam 1098 XH Amsterdam The Netherlands; ^2^ Laboratory of Plant Physiology Wageningen University & Research 6700 AA Wageningen The Netherlands; ^3^ Theoretical Biology, Department of Biology Utrecht University 3584 CH Utrecht The Netherlands

## Abstract

Endocytosis and relocalization of auxin carriers represent important mechanisms for adaptive plant growth and developmental responses. Both root gravitropism and halotropism have been shown to be dependent on relocalization of auxin transporters. Following their homology to mammalian phospholipase Ds (PLDs), plant PLDζ‐type enzymes are likely candidates to regulate auxin carrier endocytosis. We investigated root tropic responses for an Arabidopsis *pldζ1‐*KO mutant and its effect on the dynamics of two auxin transporters during salt stress, that is, PIN2 and AUX1. We found altered root growth and halotropic and gravitropic responses in the absence of PLDζ1 and report a role for PLDζ1 in the polar localization of PIN2. Additionally, irrespective of the genetic background, salt stress induced changes in AUX1 polarity. Utilizing our previous computational model, we found that these novel salt‐induced AUX1 changes contribute to halotropic auxin asymmetry. We also report the formation of “osmotic stress‐induced membrane structures.” These large membrane structures are formed at the plasma membrane shortly after NaCl or sorbitol treatment and have a prolonged presence in a *pldζ1* mutant. Taken together, these results show a crucial role for PLD*ζ1* in both ionic and osmotic stress‐induced auxin carrier dynamics during salt stress.

## Abstract 2

This work shows that both ionic and osmotic stress affect auxin carrier dynamics during salinity stress, a response that requires the action of the phospholipase PLD*ζ1*. Mathematical modelling shows that impaired cellular polarity in roots of *pldζ1* seedlings explains their different halotropism (salt avoidance) response.

## 1 INTRODUCTION

Soil conditions are one of the major decisive factors whether particular crops can be cultivated. For example, water and nutrient availability, pH, salinity, and heavy metals, but also the microbiome, all represent important factors influencing plant growth and survival (Berendsen, Pieterse, & Bakker, 2012; Kochian, Pineros, Liu, & Magalhaes, 2015; Munns & Gilliham, 2015). As a result of climate change, soil drought and salinization are encountered more frequently and are more extreme in recent years. Consequently, salt‐effected soils, present on all continents, are expected to increase, causing a decline in the amount of arable land (Wicke et al., 2011). Importantly, even moderate levels of soil salinity already cause a significant decrease in yield. Therefore, improving the salt tolerance of crops is important to secure our food supply in the near future, where we face an increasing population and decreasing amount of arable land.

Roots in a saline soil have to cope with both Na^+^ toxicity and osmotic stress. Early responses to salt include a short‐term arrest of root growth, called the quiescence phase (Geng et al., 2013). Eventually, growth reinitiates at a lower rate as before the stress (Geng et al., 2013; Julkowska & Testerink, 2015). Main root and lateral roots are affected differently by soil salinity, resulting in differences in overall root system architecture (RSA) as compared with control conditions (Julkowska et al., 2014; Julkowska et al., 2017). Besides adaptation of overall RSA to saline soils, plants are also capable of changing their direction of root growth, that is, away from salt, a phenomenon called halotropism (Galvan‐Ampudia et al., 2013).

A major player in root development, growth, and tropisms is the plant hormone auxin. Auxin maxima have been shown to promote stem cell status with somewhat lower levels supporting division (Sabatini et al., 1999), whereas auxin minima have been shown to regulate the transition from cell division to cell differentiation (Di Mambro et al., 2017). Additionally, elevation of auxin levels in elongating root cells has been shown to reduce cell expansion rate, and auxin asymmetry is the main mechanism through which growth asymmetries are established during tropisms. Tissue level auxin patterning critically depends on membrane auxin carriers of the PIN family, exporting auxins out of cells (Blilou et al., 2005), and the AUX/LAX family, importing auxin into cells (Band et al., 2014; Bennett et al., 1996). It is particularly the cell‐type‐specific expression patterns and polarity of the auxin‐exporting PINs that give rise to the so‐called polar auxin transport that shape root tip auxin patterns (Grieneisen, Xu, Marée, Hogeweg, & Scheres, 2007). Feedback of auxin on its own transporters, either through affecting PIN and AUX/LAX transcription (Laskowski et al., 2008; Vieten et al., 2005), PIN degradation (Abas et al., 2006; Kleine‐Vehn et al., 2008), or PIN membrane cycling dynamics (Paciorek et al., 2005), are assumed to play key roles in patterning auxin distribution.

Salt stress induces endocytosis, thereby enabling salt to affect auxin carriers and thus root tip auxin patterning. Clathrin‐mediated endocytosis (CME) has been proposed as the endocytic pathway involved in the internalization of auxin carriers during salt stress. Phospholipase Ds (PLDs) hydrolyse structural phospholipids, such as phosphatidylcholine, to produce phosphatidic acid (PA) that can function as lipid second messenger (for reviews, see McDermott, Wakelam, & Morris, 2004; Testerink & Munnik, 2011). PA is known to play a regulatory role during CME (Antonescu, Danuser, & Schmid, 2010). The regulation of CME by PA is putatively achieved through different modes of action. On the one hand, PA acts as a signal for cytosolic proteins to be recruited to the plasma membrane (PM; Testerink & Munnik, 2011); on the other hand, PA increases the negative curvature at the cytoplasmic leaflet of membranes thereby facilitating membrane fusion and fission (Kooijman, Chupin, & Burger, 2003; Yao & Xue, 2018).

In plants, an increase in PLD‐generated PA is measured in response to water deficit (Frank, Munnik, Kerkmann, Salamani, & Bartels, 2000; Jacob, Ritchie, Assmann, & Gilroy, 1999) and during osmotic and salt stress (Darwish, Testerink, Khalil, El‐Shihy, & Munnik, 2009). For Arabidopsis, genetic evidence for the involvement of PLDα1 and PLDδ in salt stress has been shown (Bargmann et al., 2009). Meanwhile, several candidate PA‐binding proteins have been found in the peripheral membrane fraction of salt‐stressed roots (McLoughlin et al., 2013). Treatment with an inhibitor of mammalian PLDs, 5‐fluoro‐2‐indolyl des‐chlorohalopemide (Su et al., 2009), hampered clathrin localization to the membrane (Galvan‐Ampudia et al., 2013). Consequently, PIN2 localization during salt treatment was altered in the presence of 5‐fluoro‐2‐indolyl des‐chlorohalopemide. However, *pldζ2* mutants only exhibited a weak halotropic response, indicating the possible involvement of other PLDs in the process. Yet, so far, a possible role for the other ζ‐type PLD, PLDζ1, in plant salt stress responses or tropisms has remained elusive. Inducible RNAi inhibition of PLDζ1 resulted in deformed root hairs and an altered root hair patterning (Ohashi et al., 2003). Similarly, involvement of PLDζ1 in root development during phosphate starvation has been described (Li, Qin, Welti, & Wang, 2006). Under phosphate limiting conditions, the *pldζ1/pldζ2‐*double mutant, but not the single mutants, exhibited shorter main roots and longer lateral roots. The PLDζs stand out from the other 10 plant PLDs, as they lack a Ca^2+^‐dependent C2‐binding domain, making them Ca^2+^ independent. Instead, they contain a pleckstrin homology domain and a Phox domain, similar to mammalian PLDs, which bind phosphatidylinositol lipids in membranes (Qin & Wang, 2002) and have been linked to several endocytosis and membrane recycling pathways (Donaldson, 2009).

During the halotropic response, we previously reported a salt‐induced increase in internalization and subsequent relocation of the auxin transporter PIN2 on the salt‐exposed side of the root, thus asymmetrically impacting auxin flow (Galvan‐Ampudia et al., 2013). Through a combination of computational modelling and *in planta* experiments, we subsequently demonstrated that these changes in PIN2 alone would be insufficient to generate an effective auxin asymmetry (van den Berg, Korver, Testerink, & Ten Tusscher, 2016). Auxin‐dependent regulation of AUX1 was required to amplify the auxin asymmetry. In addition, a transient increase of PIN1 that was observed in the stele was found to be essential for a rapid build‐up of this asymmetry (van den Berg et al., 2016).

We set out to investigate a putative role for PLDζ1 in salt stress responses of the root and halotropism, based on its homology to PLDζ2 and mammalian PLDs. We show that both halotropism and gravitropism are affected in the *pldζ1* mutant. In addition, we observed defects in auxin carrier localization in root epidermal cells during salt stress. Using computational modelling, we identify the PLD*ζ1*‐dependent salt‐induced relocalization of PIN2 to lateral membranes as a key event in mounting an effective halotropic response. At the same time, PLD*ζ1* appears to promote general membrane internalization induced by the osmotic component of salt. Thus, we identify PLD*ζ1* as an essential component of root tropisms and reveal their cellular role in auxin carrier and membrane relocalization in response to salinity.

## 2 METHODS

### 2.1 Plant materials and growth conditions

The wild type used was *Arabidopsis thaliana*, ecotype Columbia‐0 (Col‐0). The *pldζ1* mutant is a T‐DNA insertion line (SALK_083090). The PIN2‐GFP/*pldζ1*, AUX1‐mVenus/*pldζ1*, RabF2b‐RFPxPIN2‐GFP, SYP32‐RFPxPIN2‐GFP, RabA1e‐RFPxPIN2‐GFP, VHA1‐RFPxPIN2‐GFP, RabF2b‐RFPxPIN2‐GFP/*pldζ1*, SYP32‐RFPxPIN2‐GFP/*pldζ1*, RabA1e‐RFPxPIN2‐GFP/*pldζ1*, VHA1‐RFPxPIN2‐GFP/*pldζ1*, and PLDζ1‐YFP/*pldζ1* were created by crossing the following published lines: PIN2‐GFP (Xu & Scheres, 2005), AUX1‐mVenus (Band et al., 2014), RabF2b‐RFP, SYP32‐RFP, RabA1e‐RFP (Geldner et al., 2009), and VHA1‐RFP (Dettmer, Hong‐Hermesdorf, Stierhof, & Schumacher, 2006). Primers used for *pldζ1* genotyping are forward, tgaaaagcatggaaattttcg, and reverse, gtgatcgtctctgtctctcgc. General growth conditions on agar plates (half strength MS supplemented with 0.1% 2(*N*‐morpholino)ethanesulphonic acid buffer and 0.5% sucrose and 1% agar) were in a climate chamber with long day period (16 hr light at 130 μmol m^−2^ s^−1^) at 22°C and 70% humidity. Seeds were sterilized using 50% bleach and stratified for at least 2 days at 4°C. For soil experiments, seeds sterilized with 50% bleach were stratified in 0.1% agar in the dark for at least 2 days and then placed on sieved sowing ground. Plants were then grown were in a climate chamber with short day period (11 hr light at 130 μmol m^−2^ s^−1^) at 22°C and 70% humidity.

### 2.2 Halotropism plate assays and gravitropism plate assays

For the halotropism plate assays (both during time‐lapse imaging and long‐term halotropism assays), 10 seeds were germinated in a diagonal line on half strength MS plates. When the seedlings were 5 days old, the bottom corner (in diagonal line 0.5 cm below the root tips) of the agar was removed and replaced by control half strength MS agar without salt or half strength MS agar containing 200 mM of NaCl. For the time‐lapse experiment, the plates were placed in a climate chamber containing the time‐lapse set‐up. Here, all plates were imaged every 20 min by infrared photography. Images were then analysed using ImageJ. For the long‐term halotropism assay, a dot was placed immediately after replacing the agar and every 24 hr after the start of the treatment. After 4 days of growth, the plates were scanned, and the images were analysed using ImageJ. In the gravitropism assay, 12 plants were germinated on half strength MS plates, and after 5 days of growth, the plates were reorientated by turning 90° and placed in the climate chamber containing the time‐lapse set‐up. All plates were imaged every 20 min by infrared photography. Images were analysed using ImageJ.

### 2.3 Confocal microscopy

The images were acquired using a Nikon Ti inverted microscope in combination with an A1 spectral confocal scanning head. For all GFP fusion proteins, excitation/emission wavelengths used were 488 nm/505–555 nm. For mVenus, excitation/emission wavelengths were 514 nm/525–555 nm. For mCherry and RFP, excitation/emission wavelengths were 561 nm/570–620 nm. The analysis of the images was performed using Fiji (http://fiji.sc) software. Using a confocal microscope with a 60× objective did not yield a resolution high enough to distinguish between the apical membrane of one cell and the basal membrane of the neighbouring cell. For AUX1‐mVenus, we measured both and added them up to create the apical/basal component. All images were corrected for background signal.

Membrane intensity quantification was done using ImageJ, for all cells the average pixel intensity for the apical side (or apical/basal in the case of AUX1 and Plasma Membrane Proton ATPase 2 [PMA2]), and both lateral sides of the PM and the intracellular signal were measured by drawing a region of interest by hand.

Osmotic stress‐induced membrane structures (OSIMS) quantification was done using ImageJ; a structure was classified as an OSIM structure when it was attached to the PM but clearly on the cytosolic side of the PM and larger than 300 nm. Drug treatments were performed as follows: For filipin, seedlings were treated/stained with 10 μg ml^−1^ of filipin for 1 hr after which 120 mM of NaCl was added and plants were stressed for 60 min. For methyl‐beta‐cyclodextrin, seedlings were treated with 10 mM of methyl‐beta‐cyclodextrin for 1 hr after which 120 mM of NaCl was added and plants were stressed for 60 min.

### 2.4 Model adjustments

Our current study makes use of a previously developed model described in detail in van den Berg et al. (2016). Briefly, the model consists of a detailed two‐dimensional cross section of the Arabidopsis root tip, incorporating cell‐type and developmental zone‐specific differences in cell sizes and patterns of the auxin‐exporting PIN and auxin‐importing AUX/LAX proteins. Auxin dynamics, production, degradation, intracellular and apoplast diffusion, and across membrane fluxes are computed on a subcellular grid level resolution, whereas gene expression is simulated at the level of individual cells. In addition, we include that for AUX/LAX, gene expression is auxin dependent whereas for PIN2 membrane, occupancy levels are auxin dependent.

Relative to this earlier study, a few minor changes were implemented. First, to increase computational precision, simulations were performed with a spatial integration step of 1 rather than 2 μm, resulting in a factor 4 increase in the number of simulated grid points together constituting the root tissue. Second, to increase physiological realism, a more gradual increase in the size of cells in the early elongation zone was incorporated. For further details, we refer to our earlier study.

### 2.5 Main modelling assumptions

To apply the experimentally obtained data to our (halo)tropism model, we needed to make two key assumptions. First, salt‐induced effects on intracellular and membrane protein patterns were measured under experimental conditions in which salt was uniformly applied to the plant roots. To extrapolate these findings to halotropism where salt stress occurs (predominantly) at a single side of the root, we assumed that the experimentally observed changes in PIN2 and AUX1 occur in a similar manner but only at the salt‐exposed side of the root.

Second, in contrast to our earlier work, microscopic images were obtained using a longitudinal epidermal top view rather than a longitudinal cross section of the root. As a consequence, in addition to apical and basal membranes, radial rather than transversal lateral membrane faces were imaged. To extrapolate the thus obtained data to our halotropism model, which describes a longitudinal cross section, we assumed that PIN2 and AUX1 patterns on radial and transversal lateral membranes are similar. Support for this assumption can be derived from the observation that similar to what we previously observed for wild‐type PIN2 on transversal lateral membranes (Galvan‐Ampudia et al., 2013), wild‐type PIN2 on radial lateral membranes was observed to decrease approximately 20% in response to salt exposure.

### 2.6 Simulating *pldζ1* mutants

Our experimental data indicate that pldz1 mutants have an approximately 15% reduction in apical PIN2 levels and an approximately 10% increase in (inward) lateral PIN2 levels under control conditions. As we model standard wild‐type epidermal cells under control conditions to have a ratio of PIN2 apical:lateral of 1:0.1, we thus simulated pldz1 epidermal cells under control conditions as having a ratio of apical PIN2:lateral PIN2 of 0.85:0.11 (Figure S3). Notably, this results in a less polar PIN2 pattern, as well as a lower overall PIN2 level. These differences in PIN2 pattern result in minor changes in the default, nonhalotropic auxin pattern of *pldζ1* as compared with wild‐type roots (Figure S3).

Our experimental data indicate that in addition to changes in PIN2, cellular AUX1 patterns also change in response to salt exposure, with apical/basal levels recovering after a transient change and lateral levels showing a longer term, approximately 20% decrease. Therefore, in most halotropism simulations (indicated), we incorporated a 20% reduction of AUX1 on the lateral membranes of epidermal cells on the salt‐exposed side of the root.

### 2.7 RSA assay

*pldζ1* and wild‐type plants were germinated on half strength MS plates. Four days after germination, the seedlings were transferred to half strength MS plates with either 0 or 75 mM of NaCl. Four seedlings were transferred to each plate, resulting in 20 replicates per line per treatment. Plates were placed in the climate chamber following a randomized order. After 6 days, the plates were scanned using an Epson Perfection V800 scanner at a resolution of 400 dpi. Root phenotypes were quantified using the SmartRoot (Lobet, Pages, & Draye, 2011) plugin for ImageJ. Statistical analysis was performed in R with RStudio using a two‐way anova with Tukey's post hoc test for significance.

## 3 RESULTS

### 3.1 PLDζ1 is involved in altering the root growth direction during tropisms

**Figure 1:**
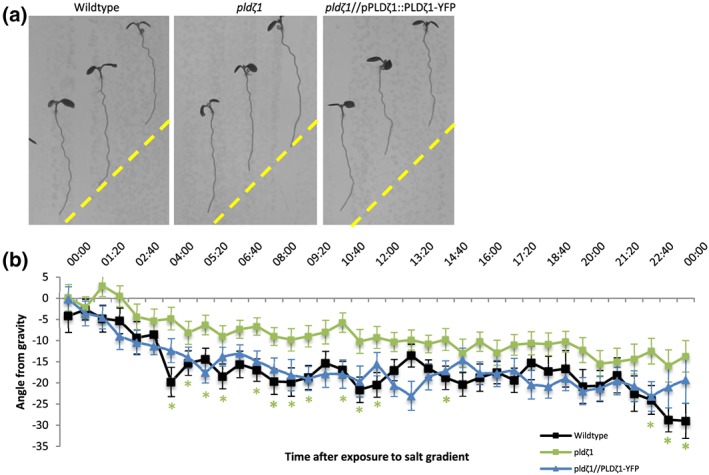
*pldζ1* mutant plant roots show a delayed halotropism response. (a) Representative images of WT (Col‐0), *pldζ1*, and *pldζ1*//pPLDζ1::PLDζ1‐YFP seedlings 24 hr after exposure to a 200‐mM NaCl gradient in a time‐lapse set‐up. Yellow dashed lines show the start of gradient. (b) Quantification of the growth angle over the first 24 hr after exposure to the 200‐mM salt gradient (combined data of two biological replicates, WT *n* = 34, *pldζ1 n* = 38, and *pldζ1*//pPLDζ1::PLDζ1‐YFP *n* = 32). Asterisks show significant differences (*P* < .05 in a univariate anova, Tukey's post hoc test); the colour of the asterisks corresponds to the line with a significant difference from wild type [Colour figure can be viewed at http://wileyonlinelibrary.com]

**Figure 2:**
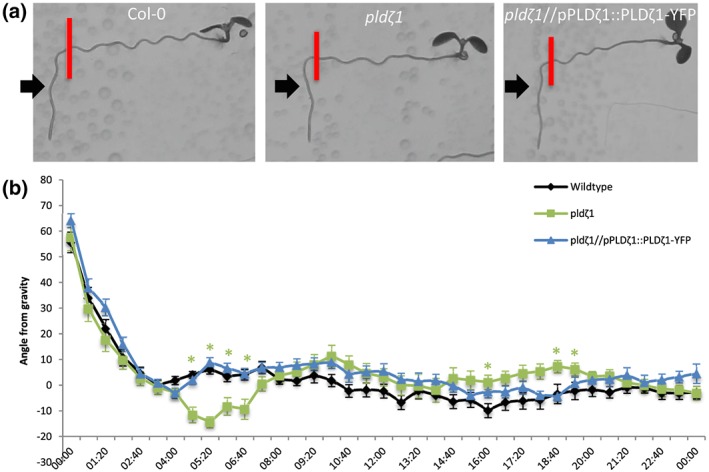
*pldζ1* roots show an exaggerated gravitropic response. (a) Representative images of WT (Col‐0), *pldζ1*, and *pldζ1*//pPLDζ1::PLDζ1‐YFP seedlings 24 hr after a 90° reorientation. Red bars show the position of the root tip immediately after reorientation. Black arrows point at the 4:00 time point. (b) Quantification of root growth angle over 24 hr after 90° reorientation (two biological replicates, WT *n* = 22, *pldζ1 n* = 24, and *pldζ1*//pPLDζ1::PLDζ1‐YFP *n* = 30). Asterisks show significant differences (*P* < .05 in a univariate anova, Tukey's post hoc test); the colour of the asterisks corresponds to the line with a significant difference from wild type [Colour figure can be viewed at http://wileyonlinelibrary.com]

To assess the putative role of PLDζ1 during halotropism, roots from a *pldζ1‐*KO mutant were followed for 1 day on vertical agar plates, containing either control medium or a salt gradient induced by 200 mM of NaCl (Galvan‐Ampudia et al., 2013). On control plates, *pldζ1* seedlings were found to display a stronger skewing effect to the right than wild type (Figure S1A,B). On salt, the *pldζ1* seedlings exhibited a slower halotropism response, and they did not reach the same angle of bending as the wild type within 24 hr (Figure 1a,b). During the long‐term halotropic response, no difference between wild type and *pldζ1* was found until Day 4, where even a slightly larger angle away from the salt gradient was observed (Figure S1C). To determine whether *pldζ1* roots were also impaired in the gravitropic response, seedlings were grown on vertical agar plates, and the root growth direction was monitored after changing the orientation of the plates by 90°. Surprisingly, *pldζ1* roots were found to display an exaggerated gravitropic response compared with the wild type (Figure 2a,b). Although wild‐type roots remained at an angle of about 10° 5 hr after reorientation, the *pldζ1* roots kept bending towards gravity. The difference was found up to 7 hr after reorientation. Skewing during normal growth was not seen until 15 hr after reorientation of the plate (Figure 2b). The slower halotropic response and the exaggerated gravitropic response were both rescued by expression of pPLDζ1::PLDζ1‐YFP in the *pldζ1* mutant background (Figures 1a,b and 2a,b). In conclusion, PLDζ1 is involved in both the halotropic and gravitropic responses.

### 3.2 AUX1 is internalized from the lateral side of the PM during salt stress

**Figure 3:**
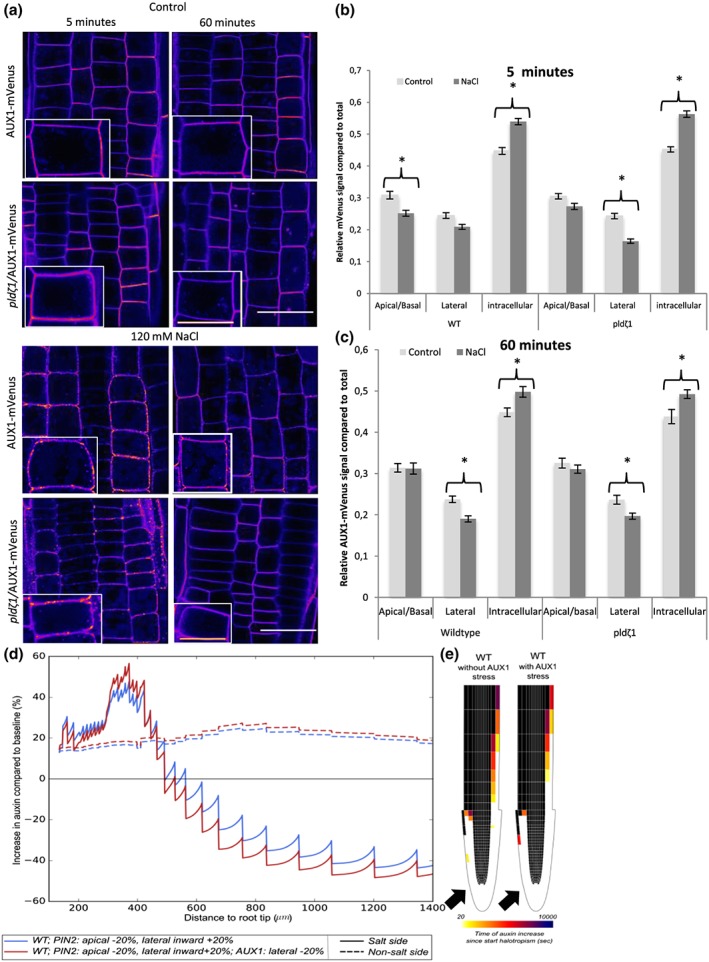
After short‐term differences in AUX1 dynamics between wild type and *pldζ* during salt stress, cells show less AUX1 on the lateral side of the PM upon salt stress after 60 min. (a) Representative pictures of AUX1‐mVenus in wild type and *pldζ1* during control and salt stress conditions (shown in fire look up table). (b) Relative AUX1‐mVenus signal abundance at the different sides of the PM compared with total signal in control and salt stress conditions after 5 min of treatment. Apical/basal is the combined signal of the adjacent apical and basal membranes because they are indistinguishable (*N* is, respectively, 88, 80, 88, and 80 cells for WT control, WT salt, *pldζ1* control, and *pldζ1* salt). Asterisks show significant differences between control and salt (*P* < .05 using *t* test in SPSS 24). (c) Relative AUX1‐mVenus signal abundance at the different sides of the PM compared with total signal in control and salt stress conditions after 60 min of treatment (*N* is, respectively, 88, 64, 96, and 80 cells for WT control, WT salt, *pldζ1* control, and *pldζ1* salt). Asterisks show significant differences between control and salt (*P* < .05 using *t* test in SPSS 24). (d) Percentage change in epidermal auxin levels at the salt and nonsalt‐exposed side of the root as a function of distance from the root tip for simulated halotropism in wild‐type roots, thus a 20% reduction in apical PIN2 and a 20% increase in lateral PIN2 in the root epidermal and root cap cells on the salt‐exposed side of the root. Changes were shown both for a situation not incorporating and a situation incorporating salt‐induced AUX1 changes. Changes in auxin levels were shown for a time point 3 hr after the start of halotropism. (e) Halotropism‐induced rerouting of auxin in the two corresponding situations. Rerouting is shown if cells experience a 10% or more increase in auxin; colour coding indicates the time after the start of halotropism when this rise in auxin levels occurs. Black arrows show the direction of the salt gradient. Scale bar = 20 μm, scale bar inlay = 10 μm [Colour figure can be viewed at http://wileyonlinelibrary.com]

Our earlier work demonstrated the importance of the interplay between auxin‐influx and auxin‐efflux carriers during halotropism (van den Berg et al., 2016). Auxin‐induced changes in AUX1 expression were shown to be crucial to amplify the auxin asymmetry required for bending. To further investigate the cellular dynamics of AUX1 during salt stress, we imaged AUX1‐mVenus during control and salt treatments over time. As expected, AUX1 showed a less polar distribution than PIN2‐GFP in control conditions (Band et al., 2014), which was similar for wild‐type and *pldζ1* roots (Figure 3a,b). After 5 min of 120 mM of NaCl, AUX1‐mVenus was observed to cluster in what appeared to be punctate structures at the PM. In wild‐type plants, this clustering occurred simultaneously with a decrease in the combined apical and basal signal (apical/basal component), whereas its combined lateral abundance did not change. In contrast, in *pldζ1* plants, mostly the lateral rather than apical/basal AUX1 abundance decreased in response to salt (Figure 3b). After 60 min of salt treatment, the apical/basal AUX1‐mVenus signal recovered to mock treatment levels for both wild type and *pldζ1*, whereas the lateral signal was still decreased, the intracellular signal increased, and less clustering at the PM was found than in the control situation (Figure 3c). These results suggest an involvement of PLD*ζ*1 in the early (minutes) salt‐induced AUX1 relocalization, but not in the long‐term AUX1 response. Additionally, these results indicate that longer salt exposure (hours) leads to a reduction in lateral AUX1 levels, irrespective of genetic background.

### 3.3 Modelling shows how salt‐induced changes in AUX1 patterning contribute to root halotropism

To investigate the effects of the altered AUX1 patterning during salt stress on halotropic auxin asymmetry, we took advantage of our previously developed computational model (van den Berg et al., 2016). Our experimental data indicated that after 60 min, roots showed restored apical/basal AUX1 levels and an approximately 20% reduction in lateral AUX1 levels. Interestingly, incorporating this 20% reduction of lateral AUX1 in our model gave rise to a 10% elevated increase in auxin at the nonsalt‐exposed side of the root (Figure 3d), whereas an even larger change in auxin decrease at the salt‐exposed side occurred. Additionally, auxin rerouting occurred in a shorter time frame, whereas the rerouting also extended further shootward (Figure 3e).

### 3.4 NaCl and sorbitol induce large membrane structures at the PM in epidermal and lateral root cap cells

**Figure 4:**
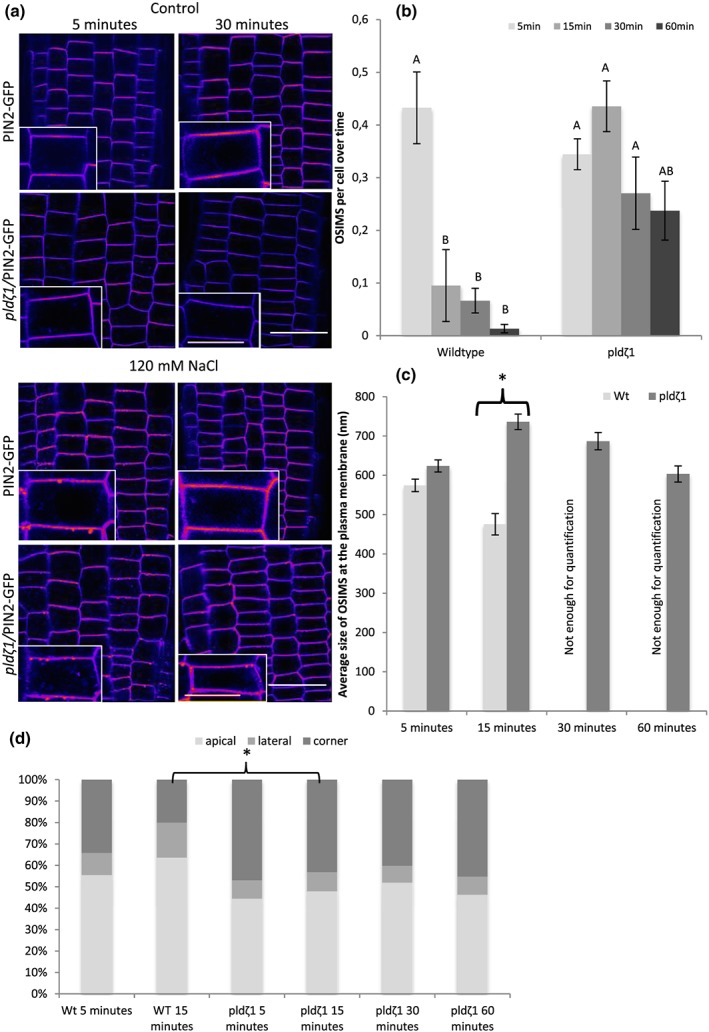
*pldζ1* mutant plants have delayed processing of OSIMS at the plasma membrane, which are bigger after 30 min and localize more often to cell corners. (a) Representative images showing PIN2‐GFP in WT and *pldζ1* in control and salt stress conditions after 5 and 60 min (shown in fire look up table). (b) Quantification of amount of osmotic stress‐induced membrane structures (OSIMS) at the plasma membrane in WT and *pldζ1* mutant plant epidermal cells in the lower elongation zone (data are from three biological replicates combined: *n* = between 350 and 500 cells per time point). (c) Average structure size in WT and *pldζ1* mutant plant epidermal cells in the lower elongation zone. Letters show significant groups, *P* < .05 in a univariate anova, Tukey's post hoc test in SPSS 24 (data are from three biological replicates combined: *n* = 50 for Col‐0 15 min, for Col‐0 5 min, and all *pldζ1 n* is between 150 and 250. For Col‐0 30 and 60 min, *N* < 10, and they were not quantified). Asterisks show significant difference between Col‐0 and *pldζ1. P* < .05 in a *t* test using SPSS 24. (d) Subcellular localization of the NaCl‐induced structures at the plasma membrane. Structures scored as localized in the corner could not be scored as either apical or lateral (the same images were used for both size and location, and the *n* is the same). Asterisks show significant differences between WT and *pldζ1* for a time point (*P* < .05 using a chi‐square test in SPSS 24). Scale bar = 20 μm, scale bar inlay = 10 μm [Colour figure can be viewed at http://wileyonlinelibrary.com]

Following the observation that AUX1 clusters to punctate structures upon root exposure to salt, we tested whether similar clustering is observed for PIN2‐GFP. Indeed, within 5 min after exposure of roots to NaCl or sorbitol, root cap and epidermal cells in wild‐type roots were observed to form large, punctate structures at the PM (Figure 4a). With an apparent size of ~600 nm (Figure 4c), these structures are too large to be any known type of PM‐associated vesicle (Heuser, 1980; Homann, 1998). In wild‐type seedlings, both number and size of these OSIMS dramatically decreased after 15 min of stress treatment (Figure 4b), and after 60 min, almost all OSIMS were gone. In contrast, the OSIMS in *pldζ1* roots build up over a much longer time (up to 15 min), whereas the average size increased to 700 nm after 15 min in the *pldζ1* roots (Figure 4c), and still half of the OSIMS remained present after 60 min of salt stress. In addition to these differences in temporal dynamics, also the subcellular localization of OSIMS showed a different pattern between wild type and *pldζ1* (Figure 4d). In wild type, ~55% of the OSIMS were located at the apical side of the membrane, whereas ~35% resided in the corner region (undistinguishable whether located on the lateral or apical side) after 5 min of salt stress. In *pldζ1* cells, less OSIMS were found at the apical side of the membrane (~45%) and more at the corners (~45%). Besides, in *pldζ1* cells, this spatial distribution did not differ between different time points, yet in wild‐type cells, even more OSIMS were found at the apical side (~64%) after 15 min of salt stress.

Next, we investigated whether a membrane protein unrelated to auxin, the PM H^+^‐ATPase PMA2, would also occur in OSIMS after salt application. Indeed, PMA2‐GFP appeared in OSIMS 5 min after salt application similar to PIN2‐GFP and AUX1‐mVenus (Figure S2A). Moreover, no significant difference in the number of OSIMS between PMA2‐ and PIN2‐GFP‐expressing lines was found 5 min after salt stress (Figure S2B). Imaging PIN2‐GFP after treatment with 240 mM of sorbitol also revealed OSIMS (Figure S2C), and no significant difference in the number of OSIMS was observed compared with salt. Similar to salt stress, these OSIMS were rarely observed after 60 min of sorbitol treatment (Figure S2D).

To test whether these OSIMS are larger forms of known endosomal structures, colocalization experiments with PIN2 and known endosomal markers were performed in wild‐type and *pldζ1* background. These include RabF2b‐RFP (ARA7) for multivesicular bodies, SYP32‐RFP for the golgi network, RabA1e‐RFP for recycling endosomes, and VHA1‐RFP for early endosomes (Dettmer et al., 2006; Geldner et al., 2009). However, none of the markers showed colocalization with the PIN2‐containing OSIMS after 5 min of NaCl treatment (Figure S3). If OSIMS represent excess membrane material, the styryl dye FM4‐64, used to label PM, should colocalize with the PIN2‐GFP found in the OSIMS. Hence, Arabidopsis seedlings expressing PIN2‐GFP were stained with FM4‐64 (Figure S4E,F). Indeed, PIN2‐GFP and FM4‐64 were found to colocalize in the OSIMS, indicating that OSIMS are of membranous origin.

To investigate whether OSIMS are part of an endocytic pathway, we tested their association with clathrin (CME) and their response to pharmacological drugs that inhibit membrane microdomain‐associated endocytosis (MMAE). For clathrin, we used a PIN2‐GFP × CLC‐mCherry line to image the subcellular localization of both proteins during early salt stress responses, and no clathrin was found to associate with OSIMS (Figure S4A,B). For MMAE, two inhibitors were tested, that is, methyl‐β‐cyclodextrin and filipin (Ovecka et al., 2010; Valitova et al., 2014), neither of which affected the formation of OSIMS (Figure S4C,D), and no colocalization was found with the fluorescent filipin (Figure S4E,F). Together, these results suggest that OSIMS represent excess membrane material but not enlarged clathrin‐coated vesicles or internalized microdomains.

### 3.5 The PIN2 auxin‐efflux carrier is more apolarly distributed in a *pldζ1* line

**Figure 5:**
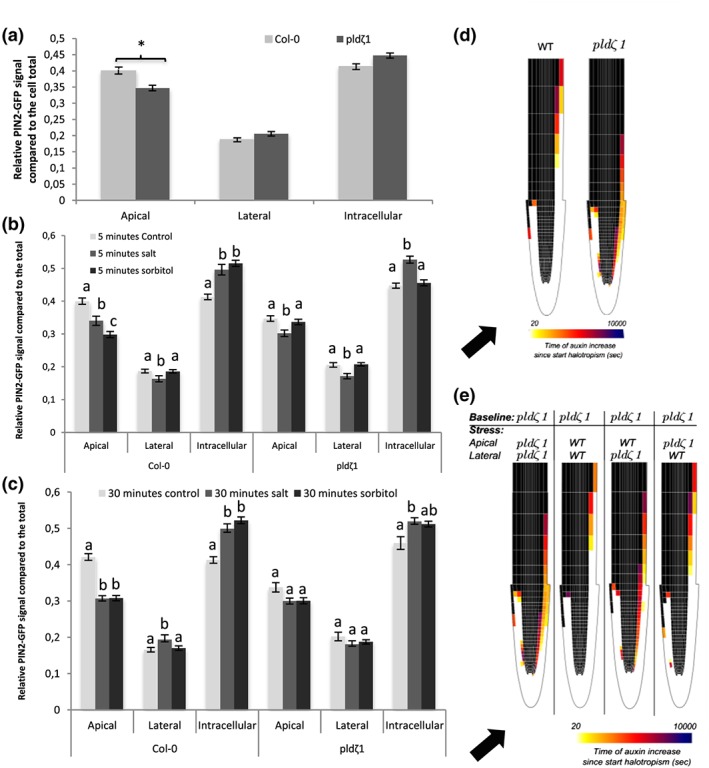
Loss of PLDζ1 results in a more apolar PIN2 distribution and altered PIN2 relocalization upon salt stress. Using a PIN2‐GFP fusion protein in both wild‐type (Col‐0) and *pldζ1* background, the intensity of the GFP signal on the apical and lateral sides of the membrane was measured next to the intracellular signal. (a) PIN2 polarity in normal conditions shows a significant lower signal at the apical membrane in the *pldζ1* background (three biological replicates, *n* = 96 cells from 12 roots). (b) Quantification of PIN2‐GFP signal at the different cell compartments after 5 min of control, salt of sorbitol treatment (three biological replicates, *n* > 80, from 10 roots or more). (c) Quantification of PIN2‐GFP signal at the different cell compartments after 30 min of control, salt of sorbitol treatment (three biological replicates, *n* > 80 from 10 roots or more). (d) Heat maps showing halotropism‐induced auxin rerouting for wild‐type and *pldζ1* settings. When a cell changes from black to coloured, this indicates an auxin increase of at least 10%. The colour indicates the timing of the auxin increase with lighter colours for short time periods. Black arrow indicates direction of the salt gradient. (e) Changes in auxin rerouting for the different stress settings in the *pldζ1* mutant. The apical or lateral distribution of PIN2 using either the *pldζ1* settings or the wild‐type stress settings in the model is indicated. When a cell changes from black to coloured, this indicates an auxin increase of at least 10%. The colour indicates the timing of the auxin increase with lighter colours for short time periods. Black arrow indicates direction of the salt gradient [Colour figure can be viewed at http://wileyonlinelibrary.com]

Next to the influx carrier AUX1, cellular polarity of the efflux carrier PIN2 has been reported to be affected by salt during the halotropism response (Galvan‐Ampudia et al., 2013). Thus, we investigated differences in the subcellular localization of PIN2 in root epidermal cells between wild type and *pldζ1* with and without salt in the root elongation zone. Under control conditions, *pldζ1* mutants exhibited a more apolar distribution of PIN2‐GFP compared with wild‐type roots, with more PIN2 at the lateral membranes and less at the apical membranes (Figures 4a and 5a). In salt‐stressed wild type, apical PIN2 abundance decreased, and intracellular PIN increased compared with total PIN2, whereas lateral PIN2 abundance increased after 30 min (Figure 5c), in accordance with our earlier work (Galvan‐Ampudia et al., 2013). As OSIMS were already formed at 5 min after treatment with salt, we next investigated early changes in polarity and found a PIN2 decrease at both apical and lateral sides of the membrane after 5 min, whereas the intracellular pool increased (Figure 5b). Thus, although initially, PIN2 at all membranes decreases in response to salt, at later stages, PIN2 is relocalized to the lateral membrane. In contrast, a membrane protein unrelated to auxin transport, that is, PMA2, displayed no such polarity change (Figure S5). The *pldζ1* mutant showed similar changes to wild type in cellular PIN2 patterning after 5 min of salt stress (Figure 5b) but failed to relocalize PIN2 to the lateral membrane at later stages after salt stress (Figure 5c).

Next, we investigated whether the differences in PIN2 polarity during NaCl treatment were salt specific or caused by osmotic shock. In order to answer this question, roots were treated with an equal osmolality of sorbitol (240 mM), and the polarity of PIN2 was determined after 5 and 30 min. Similar to 120 mM of NaCl, the apical component of the PIN2‐GFP signal in wild‐type root epidermal cells decreased after 5 min (Figure 5b). The abundance of PIN2 at the lateral sides of the membrane, however, did not change, whereas the intracellular signal increased. After 30 min, the apical, lateral, and intracellular signals all remained at the same level as the 5‐min sorbitol treatment. Thus, no significant PIN2 relocalization to the lateral sides of the PM in case of osmotic stress treatment was observed. No differences were found between the *pldζ1* mutant and wild type with regard to PIN2 internal signal and lateral relocalization in response to sorbitol (Figure 5c). On the other hand, the *pldζ1* mutant showed a different response from the wild type to sorbitol with respect to the apical PIN2 signal, which did not decrease in response to sorbitol treatment. This result indicates that osmotic stress‐induced PIN2‐GFP internalization from the apical side of the PM is impaired in the *pldζ1* (Figure 5b,c), whereas the observed PIN2 relocalization to the lateral sides of the PM upon salt stress is salt specific and PLDζ1 dependent.

### 3.6 Modelling demonstrates how differences in salt‐induced PIN2 response can delay root halotropism

Because our experimental results showed that next to salt‐induced changes in PIN2 polarity also the baseline PIN2 pattern differed between wild‐type and the *pldζ1* mutant roots, we investigated whether the observed differences in basal PIN2 patterning and those induced by salt could explain the observed response differences during halotropism. To simulate auxin patterning in the *pldζ1* mutant, we first applied the somewhat less polarized *pldζ1* mutant baseline PIN2 pattern in all lateral root cap and epidermal cells at both sides of our two‐dimensional root tip model (wild type–apical PIN2:lateral PIN2 ratio = 1:0.1; *pldζ1*–apical PIN2:lateral PIN2 ratio = 0.85:0.11; based on Figure 5a). We tested whether our model would predict differences in epidermal auxin levels in control conditions due to these changes. Apart from modestly elevated auxin levels between 250 and 500 μm from the root tip, overall root auxin patterning was largely unaffected (Figure S6). To simulate auxin patterning during halotropism in the *pldζ1* mutant, we applied the altered *pldζ*1‐type PIN2 salt responses in the higher lateral root cap and epidermal cells of only the salt‐exposed side of the root. On the basis of our data (Figure 5b), we applied a smaller reduction in apical PIN2 levels and no increase in lateral PIN2 levels as compared with wild‐type halotropism (wild‐type halotropism = apical PIN2:lateral PIN2 ratio = 0.8:0.12; *pldζ1* halotropism = apical PIN2:lateral PIN2 ratio = 0.775:0.11). The model also included the salt‐induced transient increase of PIN1, which was shown to be important for auxin asymmetry timing, and the auxin‐induced expression of AUX1, important for auxin asymmetry amplification found earlier (van den Berg et al., 2016), as well as the above discussed reduction in lateral AUX1 levels in response to salt (Figure 3c). Consistent with the experimentally observed slower halotropic response, the model predicted that *pldζ1* has a slower, less distal rerouting of auxin to the nonsalt‐exposed side of the root and achieves substantially less pronounced auxin asymmetry levels (18% vs. 27% increase at the nonsalt‐exposed side and 17% vs. 54% decrease at the salt‐exposed side; Figures 5d and S7d).

Making use of the freedom that a mathematical model offers in separating and combining conditions not easily created *in planta*, we then decided to investigate to what extent differences in *pldζ1* halotropism response depend on differences in basal PIN2 patterns and differences in salt‐induced PIN2 responses. First, we investigated the effect of the differences in baseline conditions. For this, we combined the *pldζ1* type of salt‐induced PIN2 changes with different baseline conditions: wild type, *pldζ1*, or hybrid combinations (Figure S7A). Differences in baseline PIN2 patterns appear to have a small effect on the auxin increase at the nonsalt‐exposed side, a somewhat larger effect on the auxin decrease on the salt‐exposed side, as well as a small effect on the timing of auxin rerouting (Figure S7B). The slightly larger asymmetry and rerouting speed arising in wild type as compared with *pldζ1* baseline PIN2 conditions are predominantly caused by the difference in baseline apical PIN2 levels (Figure S6A). Next, we combined *pldζ1* baseline PIN2 settings with different salt‐induced PIN2 changes: wild type, *pldζ1*, or hybrid combinations. We find that it is predominantly the increase in lateral PIN2 levels that occurs in the wild type but not *pldζ1*, under salt, that is critical for both the level of auxin asymmetry (Figures 5e and S7c) and the speed with which this asymmetry is being build‐up (Figure 5e). The higher salt‐induced apical PIN2 levels in wild type relative to *pldζ1* only play a minor role in both location and timing of the auxin asymmetry.

In conclusion, although the baseline PIN2 differences cause a mild‐slowing down and reduction of auxin rerouting during halotropism in the *pldζ1* mutant, it is predominantly the difference in salt‐induced PIN2 localization changes relative to wild type that causes a slower halotropic response due to slower, less spatially extended but foremost reduced auxin rerouting.

### 3.7 *pldζ1* root epidermal cells in the elongation zone lose turgor during the early response to mild salt stress

**Figure 6:**
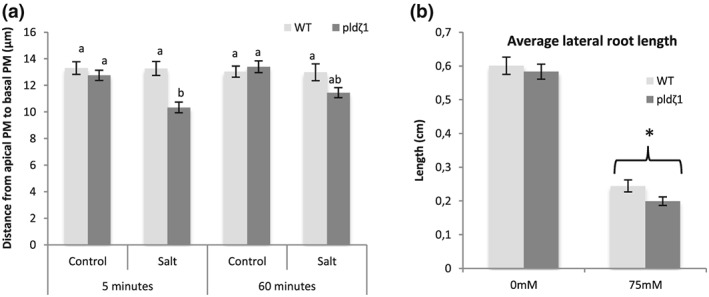
The *pldζ1* mutant exhibits a transient decrease in root epidermal protoplast size in the elongation zone and shorter lateral roots upon exposure to salt stress. (a) Five‐day‐old WT and *pldζ1* seedlings expressing pAUX1::AUX1‐mVenus were exposed to a control or 120‐mM salt treatment for either 5 or 60 min. Using confocal microscopy, cells were imaged, and distance from apical PM to basal PM was measured (data are from three biological replicates combined, for 5 min, *n* is, respectively, 88, 80, 88, and 80 cells for WT control, WT salt, *pldζ1* control, and *pldζ1* salt, and for 60 min, *n* is, respectively, 88, 64, 96, and 80 cells for WT control, WT salt, *pldζ1* control, and *pldζ1* salt). Letters show significant group determined by anova followed by Tukey's post hoc test (*P* < .05). (b) *pldζ1* seedlings have shorter average lateral root length during mild salt stress. Result are from two biological replicates, total *n* ± 40. Asterisks show significant differences between Col‐0 and *pldζ1* according to a univariate anova followed by Tukey's post hoc test with *P* < .05

As PLD*ζ*1 is involved in membrane trafficking during salt and osmotic stress, the loss of PLD*ζ*1 may lead to defective maintenance of PM surface area. In guard cells, which shrink and swell through ABA regulation, it has been shown that membrane material is internalized and remobilized back during shrinking and swelling (Meckel, Hurst, Thiel, & Homann, 2004; Shope, DeWald, & Mott, 2003). We investigated root epidermal cell shrinking in the elongation zone of *pldζ1*. Interestingly, the *pld*ζ1 mutant appears to show a mild reduction in epidermal protoplast size after 5 min of 120 mM of NaCl treatment, whereas in wild‐type cells, this concentration does not lead to plasmolysis (Figure 6a). The size reduction was only transient and was restored after 60 min (Figure 6a). Thus, the putative mild transient plasmolysis and reduced cell size in the *pldζ1* mutant suggest a slower recovery of PM surface area in response to osmotic stress.

### 3.8 Loss of PLDζ1 has a mild effect on RSA response to salt

The difference in baseline PIN2 polarity and salt‐induced AUX1 and PIN2 relocalization has been predicted by our model to result in altered auxin dynamics during halotropism in *pldζ1* roots. To determine whether the changes in auxin transporter dynamics of *pldζ1* roots result in changes in RSA on uniform salt media (no gradient), we compared root growth parameters of wild‐type and *pldζ1* seedlings transferred to agar plates containing 0 or 75 mM of NaCl (Figure S8A–E). No differences were found between wild type and *pldζ1* for main root length (Figure S8A), number of lateral roots (Figure S8B), or lateral root density (Figure S8C) in both control and mild salt stress conditions. However, *pldζ1* plants were found to have on average shorter lateral roots in salt (Figure 6b), suggesting a role for PLD*ζ1* in outgrowth of lateral roots under salt stress.

## 4 DISCUSSION

Changes in polar auxin transport in plant roots during development and in response to environmental stimuli are essential for plant survival. The endocytic pathways responsible for internalization of auxin carriers to change polar auxin transport upon stress remain elusive. For the response to salt, both clathrin‐dependent and clathrin‐independent pathways have been proposed (Baral et al., 2015; Galvan‐Ampudia et al., 2013). Mammalian PLD‐type enzymes are known to be involved in endocytosis and cell polarity, and their closest homologues in plants are PLDζs, in Arabidopsis represented by PLDζ1 and 2. Thus, PLDζs are potential candidates for regulating the internalization and cellular polarity of auxin carriers during root responses to salt stress. Previously, we demonstrated the involvement of PLDζ2 in PIN2 internalization during root halotropism, a directional growth response away from the region of the highest salt stress (Galvan‐Ampudia et al., 2013). Yet a role for PLDζ1 in salt‐induced auxin carrier internalization has never been addressed.

Our results reveal a role for PLDζ1 in tropic responses and salt stress. Furthermore, we observed the formation of large structures at the PM (OSIMS) shortly after hyperosmotic stress with NaCl or sorbitol. These OSIMS essentially disappeared in wild‐type roots within 30 min but were still found in *pldζ1* after 60 min of hyperosmotic stress. Moreover, the salt‐specific lateral relocalization of PIN2 in root epidermal cells observed in wild‐type roots was defective in a *pldζ1* mutant, whereas lateral AUX1 abundance was found to decrease irrespective of genetic background. Using our previously developed model for root halotropism simulations (van den Berg et al., 2016), we demonstrate how salt‐induced AUX1 changes could enhance halotropism‐induced auxin asymmetry. Additionally, we show that the observed PIN2 auxin carrier changes in the *pldζ1* mutant lead to a slower build‐up of auxin asymmetry during halotropism. Finally, our modelling results predict that it is predominantly the absence of an increase in lateral PIN2 in mutants as compared with wild‐type seedlings that reduces auxin asymmetry build‐up, explaining the observed delayed halotropism phenotype of the *pldζ1* mutant.

### 4.1 PLDζ1 is involved in the halotropic and gravitropic responses

For PLDζ2, a role during halotropism (Galvan‐Ampudia et al., 2013), gravitropism (Li & Xue, 2007), and hydrotropism (Taniguchi, Taniguchi, Tsuge, Oka, & Aoyama, 2010) has been shown. For PLDζ1, only a role in root hair formation (Ohashi et al., 2003) and root development during phosphate starvation had been described (Li et al., 2006), but the underlying cellular mechanisms remain unknown. Here, we report a slower halotropic response of the *pldζ1* mutant. Although *pldζ2* had a repressed halotropic response after 24 hr of growth on a salt gradient (Galvan‐Ampudia et al., 2013), for *pldζ1*, a smaller angle away from the salt gradient compared with wild type after 5–7 hr was found but no difference after 24 hr. Additionally, in contrast to the *pldζ2* mutant (Li & Xue, 2007), which showed a repressed gravitropic response, we found a normal initial yet a defective attenuation of the long‐term gravitropic response for the *pldζ1* mutant. This indicates distinct roles for PLDζ1 and PLDζ2 in the plant root during tropisms. The apparently contrasting result that the *pldζ1* mutant exhibits a repressed early‐halotropic response and an exaggerated long‐term gravitropic response might be explained by a shared importance of PIN2 relocalization during the early salt response to shift auxin to the nonsalt‐exposed side and during the late‐gravitropic response to shift back auxin to attenuate gravitropism. In this way, the dysfunctional PIN2 cycling in *pldζ1* would cause both the slow start of halotropism and the slow ending of gravitropism, underlining the importance of appropriate timing for the distinct roles of PIN2 during tropisms.

### 4.2 PLDζ1 regulates PIN2 polarity and auxin carrier polarity shifts during salt stress

It is believed that membrane protein polarity is dependent on constitutive cycling, which requires endocytosis, and PLDζ2 has been found to be involved in the endocytosis of PIN2 under normal conditions (Li & Xue, 2007). Additionally, it has been shown that PLDζ2‐derived PA regulates PIN1 polar localization through an interaction with the scaffolding A1 subunit of protein phosphatase 2A, which mediates PIN1 dephosphorylation (Gao, Chu, & Xue, 2013). We report altered polarity of PIN2 in a *pldζ1* mutant under control conditions and in addition aberrant PIN2 and AUX1 distribution during, respectively, later and initial salt stress. This is in agreement with previously shown involvement of PLDζ2 in auxin carrier relocalization during halotropism (Galvan‐Ampudia et al., 2013). Also, we found similar initial sorbitol‐induced internalization of PIN2 compared with previously reported PIN2 dynamics after a 10‐min mannitol treatment (Zwiewka, Nodzynski, Robert, Vanneste, & Friml, 2015). However, although salt induces a relocalization of PIN2 to the lateral membrane compartment, osmotic stress does not have this effect. These differences in auxin carrier relocalization during salt and osmotic treatments suggest that the relocalization during salt treatment is not due to the osmotic component of the salt stress and might explain why an osmotic gradient does not lead to a tropic (directional) response, whereas a salt gradient does (Galvan‐Ampudia et al., 2013). In support, computational modelling established that relocalization of PIN2 to the lateral side of the membrane in cells on the salt‐facing side during halotropism is required for a sufficient difference in auxin concentration between the salt‐facing and nonsalt‐facing side of the root (van den Berg et al., 2016).

PLDζ2 has been proposed to function in PIN endocytosis through the recruitment of components of the clathrin machinery to the PM by binding PLD‐derived PA (McLoughlin et al., 2013). Interaction between Clathrin Heavy Chain and Epsin‐like Clathrin Adaptor 1 and Epsin‐like Clathrin Adaptor 4 and PA was observed shortly after initiation of salt stress. The finding by McLoughlin and colleagues is consistent with other studies showing involvement of CME in internalization of auxin carriers (Galvan‐Ampudia et al., 2013; Kleine‐Vehn et al., 2010; Rakusova, Fendrych, & Friml, 2015; Shinohara, Taylor, & Leyser, 2013; Zwiewka et al., 2015) and aquaporins (Martiniere et al., 2012) during tropic responses or stress by using the CME inhibitor tyrphostin A23 (TyrA23). However, recently, it has been shown that TyrA23 treatment results in acidification of the cytoplasm (Dejonghe et al., 2016), raising serious questions on the specificity of CME inhibition by TyrA23. Other evidence pointing towards clathrin‐independent endocytosis during salt stress comes from auxin carriers that are internalized in the presence of NAA, a known inhibitor of CME (Baral et al., 2015). Moreover, ABCB19 was found to interact with PIN1 in association with sterols and sphingolipids (Titapiwatanakun et al., 2009). PIN1 was found in detergent‐resistant membrane fractions after Triton X‐100 treatment in wild‐type plants, whereas in *abcb19* mutants, PIN1 abundance in the detergent‐resistant membrane fraction was much lower. This suggests that PIN1 internalization could occur through MMAE. So, until today, no strong evidence exists for one specific endocytosis pathway induced during salt stress, and several pathways could contribute. Here, we report unknown structures arising upon salt and osmotic stress, with an apparent size of approximately 500–700 nm, containing membrane proteins, that we named OSIMS. No evidence for involvement of clathrin or microdomains in affecting these OSIMS was found.

### 4.3 Loss of PLDζ1 results in a defect in maintaining PM surface area

We describe novel OSIMS in Arabidopsis seedling roots when treated with NaCl or sorbitol. Interestingly, these osmotic stress‐induced membrane compartments were never observed detached from the PM. Therefore, we speculate that OSIMS represent excess membrane material due to osmotic stress. Invaginations or folds upon hyperosmotic stress have been observed in yeast (Morris, Winters, Coulson, & Clarke, 1983), and it has been proposed that in stomatal guard cell protoplasts, changes in membrane surface area are associated with removal and incorporation of membrane material (Homann, 1998). The fact that OSIMS were only observed in the first 30 min after NaCl or sorbitol treatment in wild‐type plants and that Arabidopsis epidermal root cells have been shown to recover turgor pressure within 40 min after hyperosmotic stress (Shabala & Lew, 2002) supports this theory. To test whether OSIMS are enlarged, growing vesicles, we performed colocalization experiments with markers for known endocytosis pathways. The OSIMS were not found to colocalize with CLC2 (marker for clathrin‐coated vesicles) or filipin (a marker for membrane microdomains), suggesting that they are not enlarged vesicles. Consistently, two drugs commonly used to inhibit MMAE did not affect the presence of OSIMS. These results support the hypothesis that the OSIMS are excess PM material induced by hyperosmotic treatment. Additionally, AUX1‐mVenus was found to form punctate structures in the PM, which appear to reside longer in the PM in wild‐type cells than *pldζ1* cells, indicating that besides OSIMS, other structures may influence auxin carrier dynamics during osmotic stress. The observation that loss of PLDζ1 leads to a slight decrease in protoplast size of root epidermal cells indicates slow recovery of PM surface area after osmotic stress and thus involvement of PLDζ1 in membrane fission or restoration of osmotic potential.

### 4.4 Loss of PLDζ1 affects root remodelling during salt stress

Recently, the growth rate of a *pldζ1* mutant in soil containing 75 mM of NaCl was found to be more affected than in wild type (Ben Othman et al., 2017), but root architecture was not addressed in this study. Different strategies have been described for Arabidopsis accessions in RSA adaptations to salinity (Julkowska et al., 2014). For the wild‐type accession Col‐0, it is known that lateral root length contribution towards total root size increases upon an increase in salt concentration (Julkowska et al., 2014). Thus, a shorter main root and longer laterals might be a response to cope with higher salinity. In the current study, we observed in the *pldζ1* mutant a decreased lateral root length to main root length ratio during mild salt stress. Because this represents a reversal of the wild‐type salinity response, this supports the idea of defective remodelling in this mutant and could underlie the observed slightly reduced coping capacity of *pldζ1* plants in saline soil (Ben Othman et al., 2017).

### 4.5 Lateral AUX1 decrease, PLDζ1, and OSIMS affect the salt response in Arabidopsis roots

Concluding, we report salt‐induced changes in AUX1 localization, and through mathematical modelling, we predict that the observed decrease of lateral AUX1 enhances auxin asymmetry during halotropism. Moreover, this work confirms a role for PLDζ1 in halotropic and gravitropic responses. A *pldζ1* mutant exhibited both permanently altered PIN2 polarity and changes in salt‐induced PIN2 relocalization. Simulations in our updated mathematical root model show how these differences can result in delayed and weaker build‐up of auxin asymmetry in the *pldζ1* mutant, thus explaining its halotropism phenotype. Finally, we discovered novel OSIMS. The hampered processing of OSIMS in the *pldζ1* mutant coincides with the absence of PIN2 relocalization to the lateral side of the PM during salt stress and thus indicates a role for OSIMS in the auxin carrier dynamics during salt stress. Together, these results further our knowledge on auxin carrier internalization and relocalization during the salt stress response.

## AUTHOR CONTRIBUTIONS

R.A.K., T.v.d.B., K.H.W.J.t.T., and C.T. conceptualized the study. T.v.d.B. and K.H.W.J.t.T. contributed to the software. R.A.K., A.J.M., and T.v.d.B. contributed to the investigation of the study. C.S.G.A. contributed to the resources. R.A.K. and C.T. wrote the original draft. All authors reviewed and edited the study. C.T. and K.H.W.J.t.T contributed to the supervision and funding acquisition.

## Supporting information


**Figure S1:**
Long term effects on halotropic response show delayed attenuation of the halotropic response. (A) Images showing 5 days of growth of WT and the *pldζ1* mutant on gradient plates with 0 mM or 200 mM of NaCl. (B,C) Quantification of halotropism plate assay over multiple days shows more skewing of *pldζ1* mutant roots in control conditions (B)(3 biological replicates: WT n = 68, *pldζ1* n = 62) and more avoidance after 4 days of growth on a salt medium (C) (3 biological replicates: WT n = 67, *pldζ1* n = 66). Asterisks show significant differences, an univariate ANOVA was used followed by a Tukey post‐hoc test (p < 0.05)

**Figure S2:**
OSIMS contain multiple plasma membrane proteins and are induced by osmotic stress. (A) Comparison of the amount of OSIMS in a PIN2‐GFP, AUX1‐mVneus and PMA2‐GFP line after 5 minutes of a 120 mM salt stress. No significant differences were found using a univariate ANOVA with Tukey post hoc in SPSS 24. (B) Representative images of OSIMS in Arabidopsis roots expressing PIN2‐GFP, AUX1‐mVenus and PMA2‐GFP after a 5 minute 120 mM NaCl treatment. (C) Representative pictures of PIN2‐GFP sub‐cellular localization after 5 or 60 minutes of 240 mM Sorbitol treatment. Inlays show an enlargement of one cell. (D) Quantification of the average number of OSIM structures per cell after 5 and 60 minutes of 240 mM Sorbitol treatment, salt treatment data is shown for comparison. No significant differences using a univariate ANOVA followed by a Tukey post‐hoc test (p < 0.05) were found for either time point. Scale bar = 10 μm in (A), 20 μm in (C) and 10 μm in the inlay.

**Figure S3:**
OSIMS do not co‐localize with known endosomal markers. Representative images showing PIN2‐GFP in wildtype and *pldζ1* background in combination with either RabF2b‐RFP (ARA7) for multi‐vesicular bodies, SYP32‐RFP for the golgi network, RabA1e‐RFP for recycling endosomes and VHA1‐RFP for early endosomes after 5 minutes of salt stress. Yellow lines are the lines used for the profile plot through the OSIMS. Green line shows PIN2‐GFP signal and the magenta line shows the endosomal marker RFP signal. No OSIMS are found in control conditions so no profile plots are shown. Scale bar = 20 μm

**Figure S4:**
OSIMS do not co‐localize with clathrin light chain and are not inhibited by membrane micro‐domain inhibiting drugs. (A) Representative image of a *pldζ1* line expressing PIN2‐GFP and CLC‐mCherry during a salt treatment. (B) Profile plot of the yellow line in (A) showing PIN2‐GFP and CLC‐mCherry intensities just below the apical side of the PM crossing through one OSIMS. (C) Representative images showing a *pldζ1* line expressing PIN2‐GFP during salt stress with either no drug, 10ug/ml Filipin or 10 mM of Methyl‐beta‐Cyclodextrin. (D) Quantification of average number of OSIMS per cell during no drug treatment (n = 12), Filipin treatment (n = 8) and M‐b‐CD treatment (N = 14). No significant differences were found (p < 0.05 in a univariate ANOVA, Tukey post hoc using SPSS 24). (E) Enlargements of one cell expressing PIN2‐GFP and stained with fm4‐64 and filipin showing one OSIMS. (F) Profile plot the yellow line in (E) showing the OSIMS contains PIN2‐GFP, fm4‐64 but not filipin. Scale bar = 10 μm in (A), 20 μm in (B) and 10 μm in (C).

**Figure S5:**
No change in PMA2 cellular polarity during salt stress. PMA2‐GFP expressing seedlings were treated with either control or salt containing (120 mM NaCl) medium. After 5 minutes the PMA2‐GFP signal was measured on the apical and lateral side of the PM as well as the intracellular signal. No differences between the treatments were observed, an univariate ANOVA followed by a Tukey post‐hoc was used. N = 32 cells from 2 biological replicates.

**Figure S6:**
Comparison of default, non‐halotropic simulated auxin patterns in wildtype and *pldζ1* plants. (A) Epidermal auxin levels as a function of distance from the root tip in wildtype and *pldζ1* plants. (B) Model PIN pattern, highlighting differences between wildtype and *pldζ1* in PIN2 patterning under both baseline and salt stress conditions. (C) Simulated DR5 auxin marker pattern for wildtype and *pldζ1* plants. Colors depict auxin concentration.

**Figure S7:**
Effect of baseline differences and salt induced differences in PIN2 polarity on epidermal auxin levels during halotropism. (A) Changes in epidermal auxin levels when combining *pldζ1* type halotropic PIN2 patterning changes with different baseline PIN2 patterns, wildtype, *pldζ1* or hybrid combinations over the lower part of the root after 24 hours. For comparison purposes also the auxin dynamics in wildtype plants are shown. (B) Changes in auxin rerouting for the different settings. A colored cell has an auxin increase of at least 10% the different colors depict different times. (C) Changes in epidermal auxin levels when combining *pldζ1* baseline PIN2 patterns with different salt induced changes in PIN2 patterning, wildtype, *pldζ1* or hybrid combinations. For comparison purposes also the auxin dynamics in wildtype plants are shown over the lower part of the root after 24 hours. (D) Changes in epidermal auxin levels for *pldζ1* and wildtype plants over the lower part of the root during halotropism after 24 hours.

**Figure S8:**
*pldζ1* has shorter lateral roots and both main root and lateral roots grow in a different angle during salt stress. pldζ1 and WT plants were germinated on half strength MS plates. Four days after germination the seedlings were transferred plates with either 0 mM or 75 mM NaCl. Four seedlings were transferred to each plate, after six days of growth roots were analyzd. *pldζ1* has no change in main root length (A), number of lateral roots (B) or lateral root density (C). Significant differences that are found are;, main root direction (D), and lateral root direction (E). Result are from 2 biological replicates, total n ± 40. Asterisks show significant differences between Col‐0 and *pldζ1* according to an univariate ANOVA followed by a Tukey post hoc test with p < 0.05.
