# Supplementary figures and images for: Halotropism requires phospholipase Dζ1‐mediated modulation of cellular polarity of auxin transport carriers
Source: Plant Cell Environ. 2019 Oct 2;43(1):143–58. doi: 10.1111/pce.13646 (PMC6972530; doi:10.1111/pce.13646)

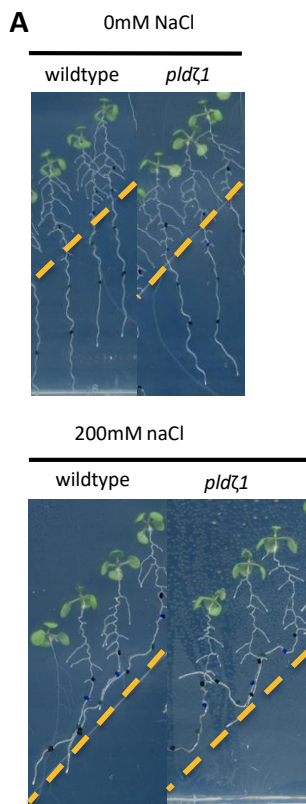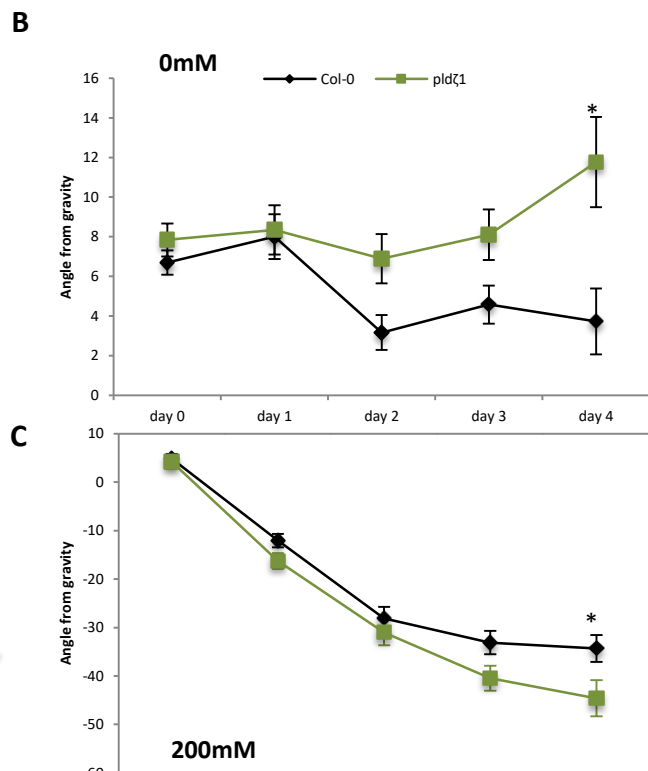

Supplement: Supplementary file 1 — Figure S1: Long term effects on halotropic response show delayed attenuation of the halotropic response. (A) Images showing 5 days of growth of WT and the pldζ1 mutant on gradient plates with 0 mM or 200 mM of NaCl. (B,C) Quantification of halotropism plate assay over multiple days shows more skewing of pldζ1 mutant roots in control conditions (B)(3 biological replicates: WT n = 68, pldζ1 n = 62) and more avoidance after 4 days of growth on a salt medium (C) (3 biological replicates: WT n = 67, pldζ1 n = 66). Asterisks show significant differences, an univariate ANOVA was used followed by a Tukey post‐hoc test (p < 0.05) [file PCE-43-143-s001.pdf]

**A**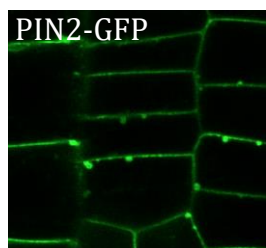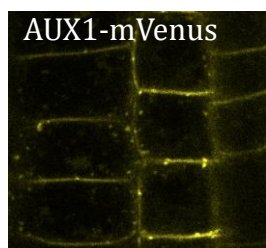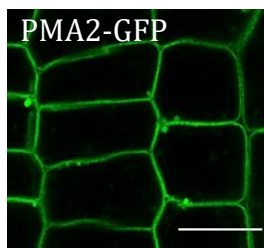**B**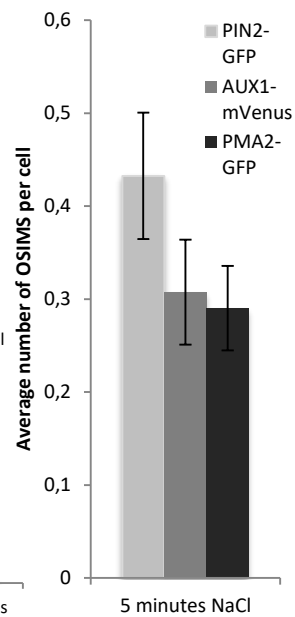**C**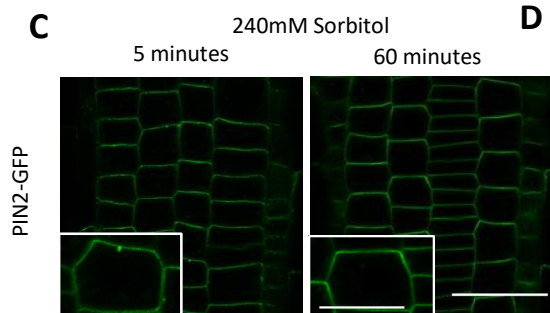**D**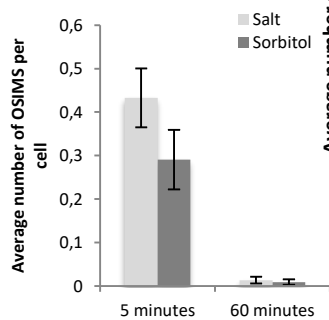

Supplement: Supplementary file 2 — Figure S2: OSIMS contain multiple plasma membrane proteins and are induced by osmotic stress. (A) Comparison of the amount of OSIMS in a PIN2‐GFP, AUX1‐mVneus and PMA2‐GFP line after 5 minutes of a 120 mM salt stress. No significant differences were found using a univariate ANOVA with Tukey post hoc in SPSS 24. (B) Representative images of OSIMS in Arabidopsis roots expressing PIN2‐GFP, AUX1‐mVenus and PMA2‐GFP after a 5 minute 120 mM NaCl treatment. (C) Representative pictures of PIN2‐GFP sub‐cellular localization after 5 or 60 minutes of 240 mM Sorbitol treatment. Inlays show an enlargement of one cell. (D) Quantification of the average number of OSIM structures per cell after 5 and 60 minutes of 240 mM Sorbitol treatment, salt treatment data is shown for comparison. No significant differences using a univariate ANOVA followed by a Tukey post‐hoc test (p < 0.05) were found for either time point. Scale bar = 10 μm in (A), 20 μm in (C) and 10 μm in the inlay. [file PCE-43-143-s002.pdf]

Wildtype background

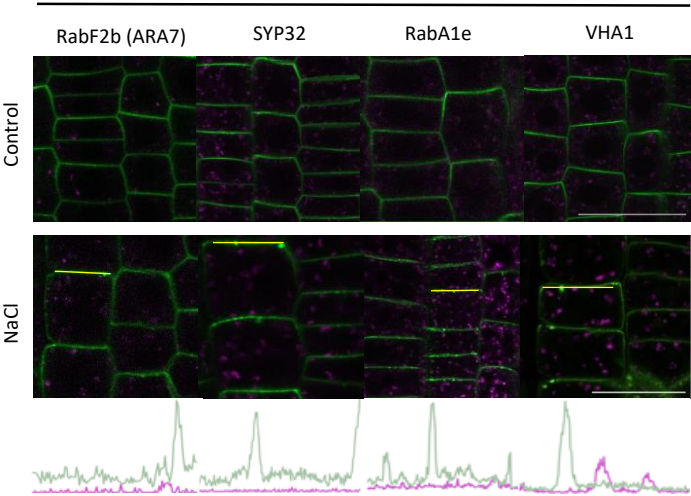

*pldζ1* background

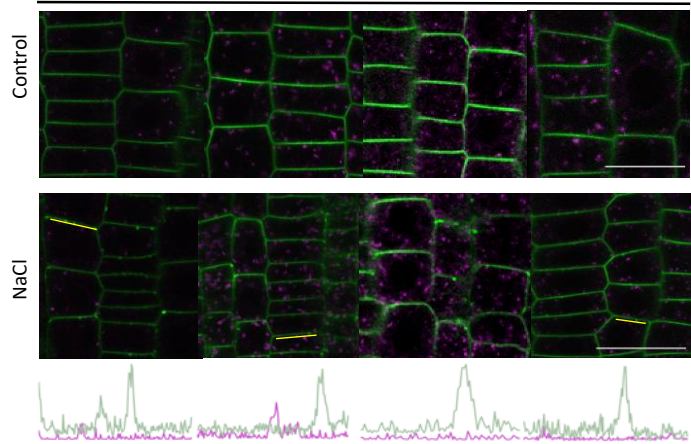

Supplement: Supplementary file 3 — Figure S3: OSIMS do not co‐localize with known endosomal markers. Representative images showing PIN2‐GFP in wildtype and pldζ1 background in combination with either RabF2b‐RFP (ARA7) for multi‐vesicular bodies, SYP32‐RFP for the golgi network, RabA1e‐RFP for recycling endosomes and VHA1‐RFP for early endosomes after 5 minutes of salt stress. Yellow lines are the lines used for the profile plot through the OSIMS. Green line shows PIN2‐GFP signal and the magenta line shows the endosomal marker RFP signal. No OSIMS are found in control conditions so no profile plots are shown. Scale bar = 20 μm [file PCE-43-143-s003.pdf]

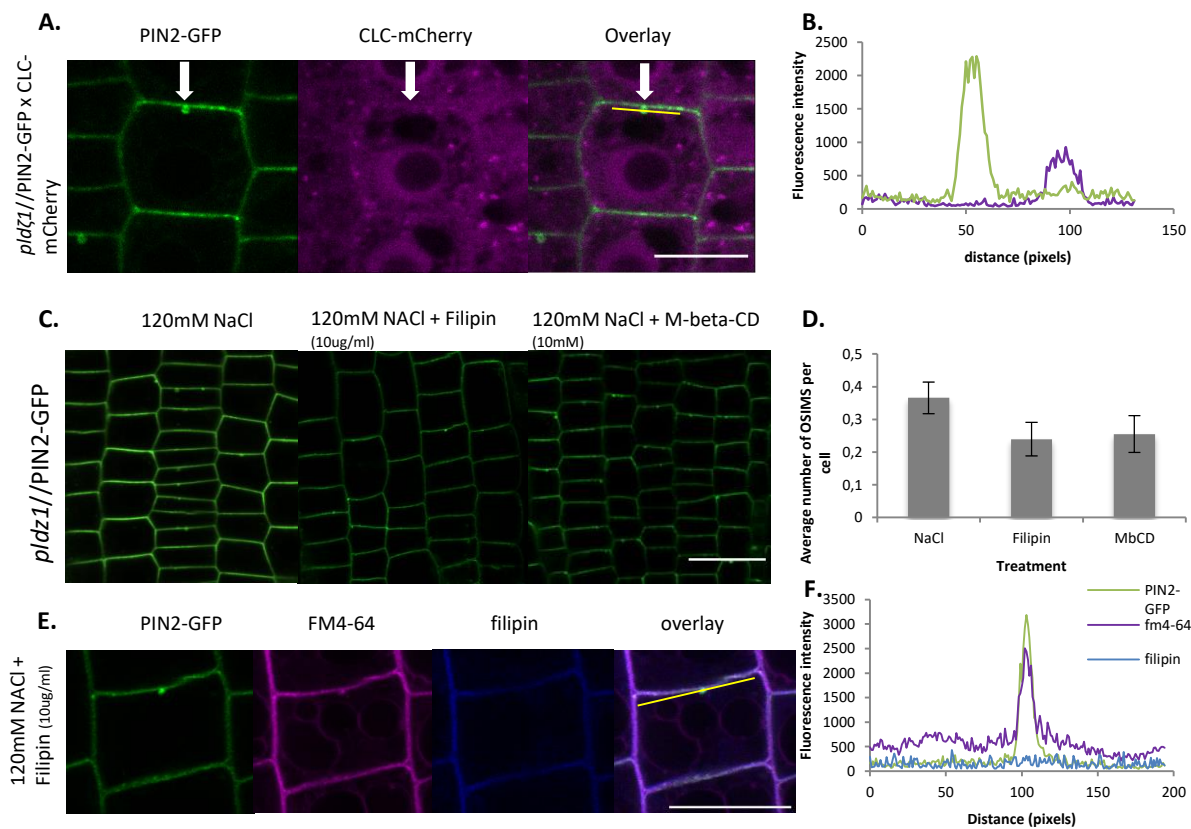

Supplement: Supplementary file 4 — Figure S4: OSIMS do not co‐localize with clathrin light chain and are not inhibited by membrane micro‐domain inhibiting drugs. (A) Representative image of a pldζ1 line expressing PIN2‐GFP and CLC‐mCherry during a salt treatment. (B) Profile plot of the yellow line in (A) showing PIN2‐GFP and CLC‐mCherry intensities just below the apical side of the PM crossing through one OSIMS. (C) Representative images showing a pldζ1 line expressing PIN2‐GFP during salt stress with either no drug, 10ug/ml Filipin or 10 mM of Methyl‐beta‐Cyclodextrin. (D) Quantification of average number of OSIMS per cell during no drug treatment (n = 12), Filipin treatment (n = 8) and M‐b‐CD treatment (N = 14). No significant differences were found (p < 0.05 in a univariate ANOVA, Tukey post hoc using SPSS 24). (E) Enlargements of one cell expressing PIN2‐GFP and stained with fm4‐64 and filipin showing one OSIMS. (F) Profile plot the yellow line in (E) showing the OSIMS contains PIN2‐GFP, fm4‐64 but not filipin. Scale bar = 10 μm in (A), 20 μm in (B) and 10 μm in (C). [file PCE-43-143-s004.pdf]

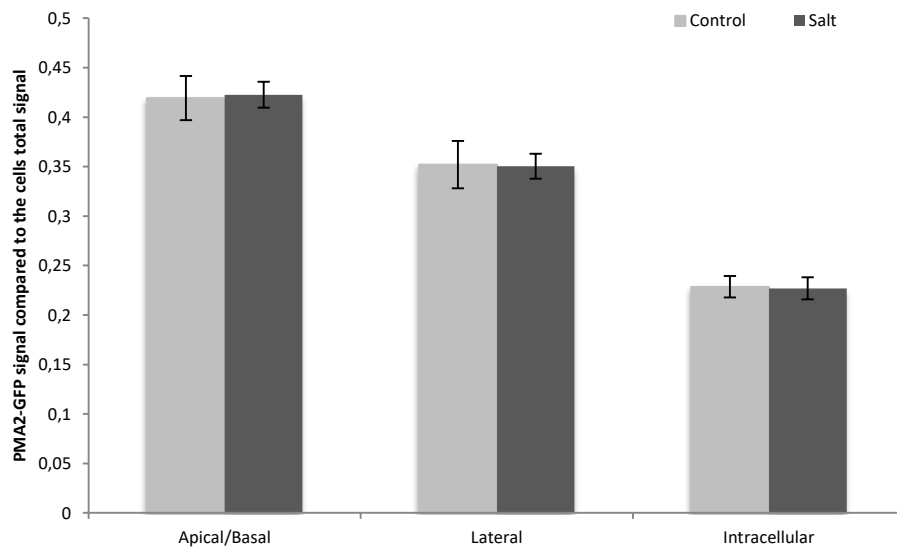

Supplement: Supplementary file 5 — Figure S5: No change in PMA2 cellular polarity during salt stress. PMA2‐GFP expressing seedlings were treated with either control or salt containing (120 mM NaCl) medium. After 5 minutes the PMA2‐GFP signal was measured on the apical and lateral side of the PM as well as the intracellular signal. No differences between the treatments were observed, an univariate ANOVA followed by a Tukey post‐hoc was used. N = 32 cells from 2 biological replicates. [file PCE-43-143-s005.pdf]

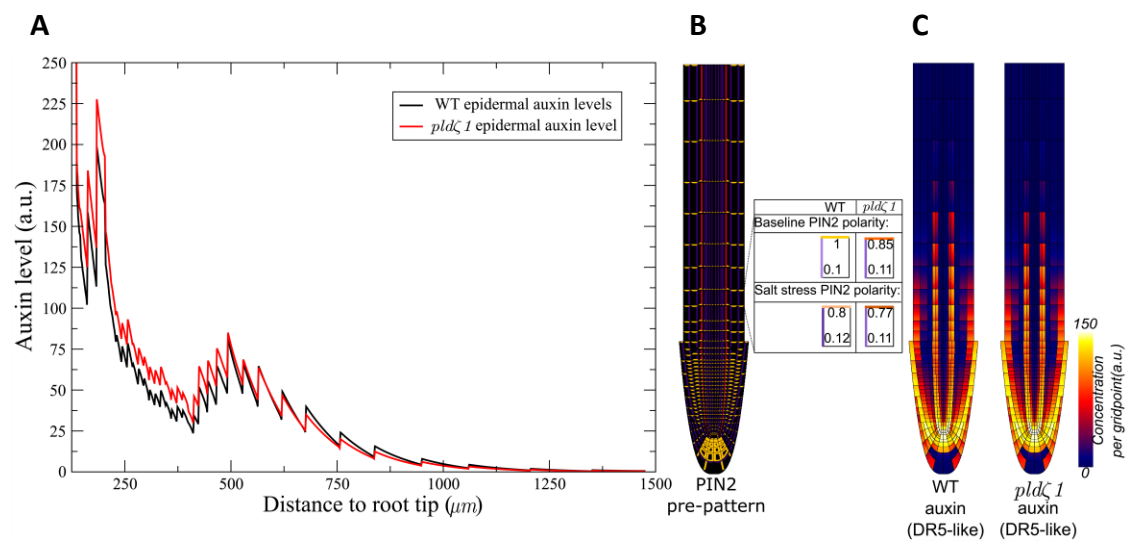

Supplement: Supplementary file 6 — Figure S6: Comparison of default, non‐halotropic simulated auxin patterns in wildtype and pldζ1 plants. (A) Epidermal auxin levels as a function of distance from the root tip in wildtype and pldζ1 plants. (B) Model PIN pattern, highlighting differences between wildtype and pldζ1 in PIN2 patterning under both baseline and salt stress conditions. (C) Simulated DR5 auxin marker pattern for wildtype and pldζ1 plants. Colors depict auxin concentration. [file PCE-43-143-s006.pdf]

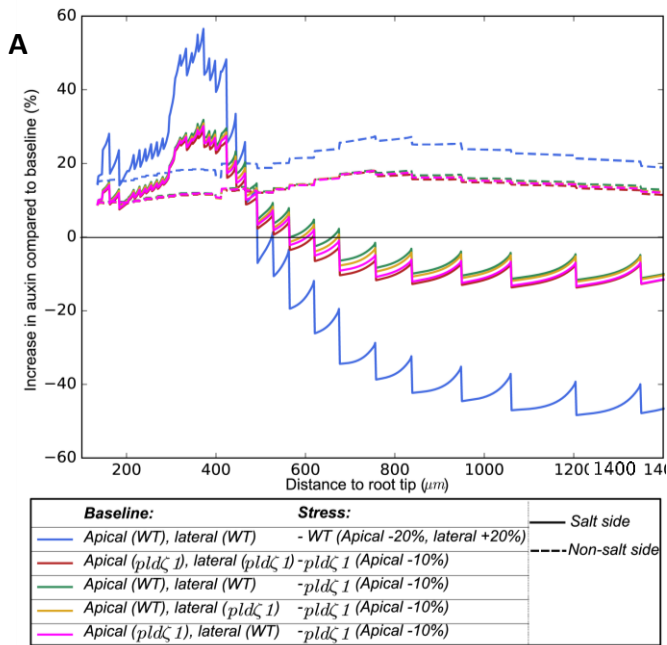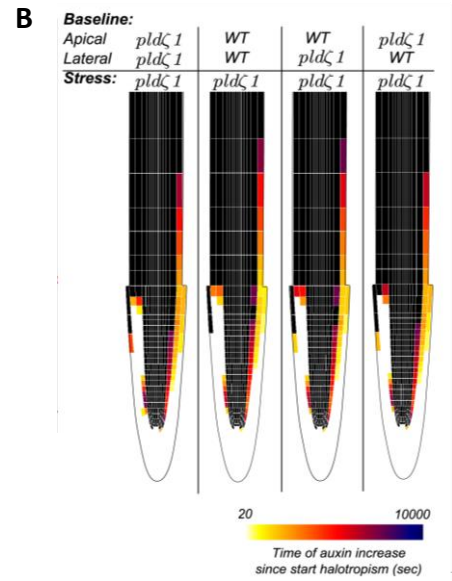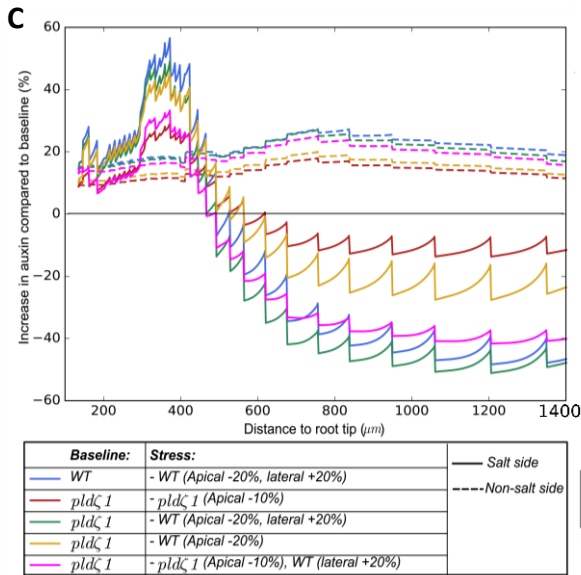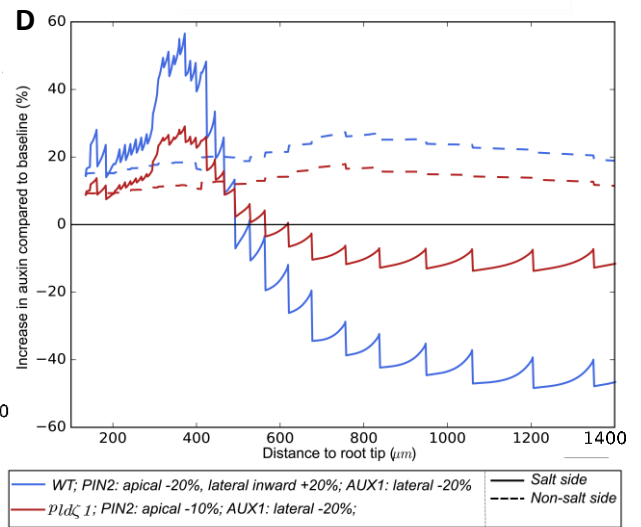

Supplement: Supplementary file 7 — Figure S7: Effect of baseline differences and salt induced differences in PIN2 polarity on epidermal auxin levels during halotropism. (A) Changes in epidermal auxin levels when combining pldζ1 type halotropic PIN2 patterning changes with different baseline PIN2 patterns, wildtype, pldζ1 or hybrid combinations over the lower part of the root after 24 hours. For comparison purposes also the auxin dynamics in wildtype plants are shown. (B) Changes in auxin rerouting for the different settings. A colored cell has an auxin increase of at least 10% the different colors depict different times. (C) Changes in epidermal auxin levels when combining pldζ1 baseline PIN2 patterns with different salt induced changes in PIN2 patterning, wildtype, pldζ1 or hybrid combinations. For comparison purposes also the auxin dynamics in wildtype plants are shown over the lower part of the root after 24 hours. (D) Changes in epidermal auxin levels for pldζ1 and wildtype plants over the lower part of the root during halotropism after 24 hours. [file PCE-43-143-s007.pdf]

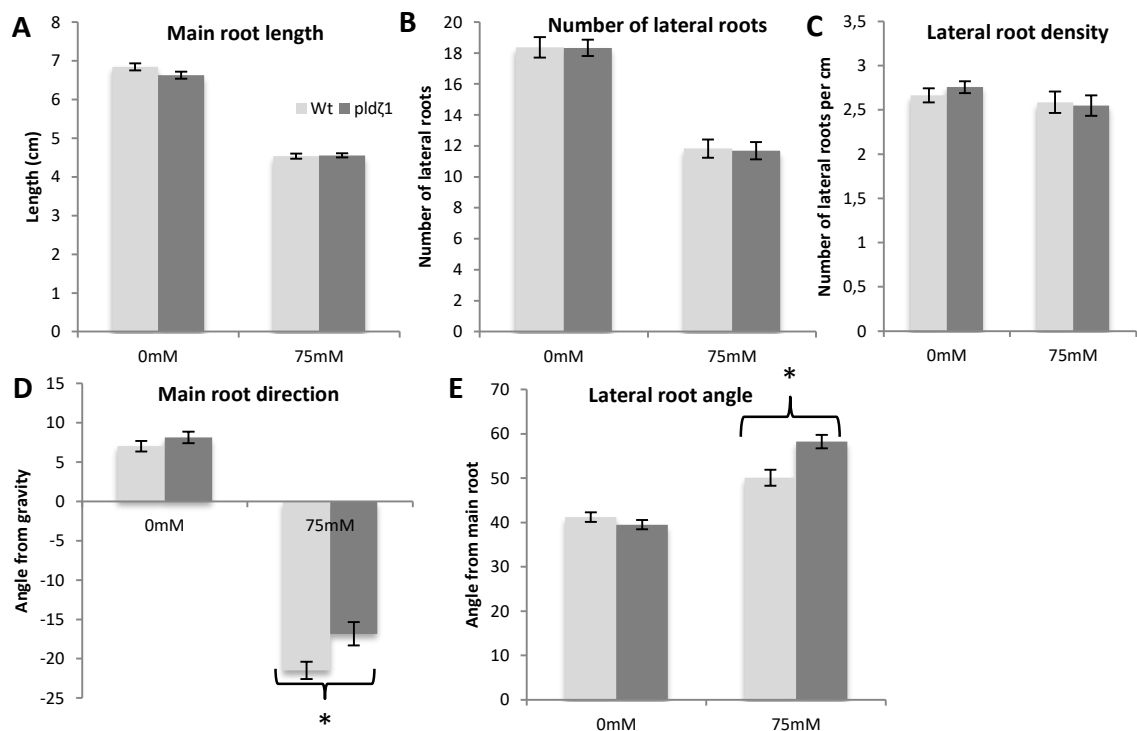

Supplement: Supplementary file 8 — Figure S8: pldζ1 has shorter lateral roots and both main root and lateral roots grow in a different angle during salt stress. pldζ1 and WT plants were germinated on half strength MS plates. Four days after germination the seedlings were transferred plates with either 0 mM or 75 mM NaCl. Four seedlings were transferred to each plate, after six days of growth roots were analyzd. pldζ1 has no change in main root length (A), number of lateral roots (B) or lateral root density (C). Significant differences that are found are;, main root direction (D), and lateral root direction (E). Result are from 2 biological replicates, total n ± 40. Asterisks show significant differences between Col‐0 and pldζ1 according to an univariate ANOVA followed by a Tukey post hoc test with p < 0.05. [file PCE-43-143-s008.pdf]
